# “We know it’s labour pain, so we don’t do anything”: healthcare provider’s knowledge and attitudes regarding the provision of pain relief during labour and after childbirth

**DOI:** 10.1186/s12884-018-2076-7

**Published:** 2018-11-14

**Authors:** Mary McCauley, Valentina Actis Danna, Dorah Mrema, Nynke van den Broek

**Affiliations:** 1 Centre for Maternal and Newborn Health, Liverpool School of Tropical Medicine, Pembroke Place, Liverpool, L3 5QA UK; 2 Kilimanjaro Christian Medical Centre, Moshi, Kilimanjaro Tanzania

**Keywords:** Pain relief, Analgesia, Maternity care, Quality of care, Labour, Childbirth, Healthcare providers

## Abstract

**Background:**

Most women experience pain during labour and after childbirth. There are various options, both pharmacological and non-pharmacological, available to help women cope with and relieve pain during labour and after childbirth. In low resource settings, women often do not have access to effective pain relief. Healthcare providers have a duty of care to support women and improve quality of care. We investigated the knowledge and attitudes of healthcare providers regarding the provision of pain relief options in a hospital in Moshi, Tanzania.

**Methods:**

Semi-structured key informant interviews (*n* = 24) and two focus group discussions (*n* = 10) were conducted with healthcare providers (*n* = 34) in Tanzania. Transcribed interviews were coded and codes grouped into categories. Thematic framework analysis was undertaken to identify emerging themes.

**Results:**

Most healthcare providers are aware of various approaches to pain management including both pharmacological and non-pharmacological options. Enabling factors included a desire to help, the common use of non-pharmacological methods during labour and the availability of pharmacological pain relief for women who have had a Caesarean section. Challenges included shortage of staff, lack of equipment, no access to nitrous oxide or epidural medication, and fears regarding the effect of opiates on the woman and/or baby. Half of all healthcare providers consider labour pain as ‘natural’ and necessary for birth and therefore do not routinely provide pharmacological pain relief. Suggested solutions to increase evidence-based pain management included: creating an enabling environment, providing education, improving the use of available methods (both pharmacological and non-pharmacological), emphasising the use of context-specific protocols and future research to understand how best to provide care that meets women’s needs.

**Conclusions:**

Many healthcare providers do not routinely offer pharmacological pain relief during labour and after childbirth, despite availability of some resources. Most healthcare providers are open to helping women and improving quality of pain management using an approach that respects women’s culture and beliefs. Women are increasingly accessing care during labour and there is now a window of opportunity to adapt and amend available maternity care packages to include comprehensive provision for pain relief (both pharmacological and non-pharmacological) as an integral component of quality of care.

## Background

Most women experience pain during labour and after childbirth. The World Health Organization (WHO) includes pain management as a standard of quality of care, highlighting that all aspects of health care should be given timely, appropriately, and should respect a woman’s choice, culture and needs [1].

The Sustainable Development Goal three highlights health and well-being, and the Global Strategy for Women’s, Children’s and Adolescent’s Health emphasises that all women have the right to, and should obtain, the highest attainable standard of health, including physical and psychological care [2, 3].

The severity of pain and its detrimental impact on the health and well-being of mothers during labour and after childbirth has resulted in health policy development in many high-income countries such as the United Kingdom (UK), where pain relief options (both pharmacological and non-pharmacological) are routinely discussed during antenatal contacts and then offered during intrapartum care by a trained healthcare provider, as per the individual woman’s choice [4–6].The provision of effective routine pain relief results in a more positive experience of labour and childbirth for the woman [4, 7].

Women report feeling empowered and in control when they have been enabled to make informed decisions [8], including the choice of how to cope with and alleviate their pain during labour and after childbirth [9]. In low resource settings, pain management options (especially pharmacological options) are not well-established and the provision of pain relief options often depends on the health system capacity, the knowledge and attitudes of healthcare providers and availability and cost of medications [10–14]. Healthcare providers can play a positive role by educating women about the options available and supporting their choice in coping with labour pain; or a negative role by demonstrating disrespectful care, withholding care and lack of communication [11, 12, 14] .

Globally, 78% of women give birth with the assistance of a skilled birth attendant [13]. With increasing numbers of women accessing maternity care in low resource settings, there are many potential opportunities for healthcare providers to improve the quality of care for women. There are international policies and guidelines to increase quality of care (including pain management) but, at present, there is little practical implementation. It is imperative that healthcare providers are enabled to provide respectful maternity care that goes beyond the provision of basic emergency care and includes pain management (especially pharmacological) as a component of health and well-being during labour and after childbirth [3, 13].

There have been several studies regarding pain management during labour and after childbirth in low resource settings to assess women’s knowledge and views on the acceptability and use of different pain management options during labour [15–24]. However, there is lack of research exploring how the perspectives of healthcare providers can affect the provision of pain relief options and the quality of care experience for women during labour and after childbirth.

This study sought to assess the knowledge and attitudes regarding the provision of pain relief during labour and after childbirth among healthcare providers who provide routine maternity care in Tanzania. In addition, we explored enabling factors and barriers as well as healthcare providers’ recommendations regarding how pain relief options (both pharmacological and non-pharmacological) could be integrated as important components of respectful maternity care in low resource settings.

## Materials and methods

### Study design and setting

A qualitative descriptive design was used. Data collection was conducted between May and June 2017, using semi-structured key informant interviews (KII) and focus group discussions (FGD). Participants were recruited from the Departments of Obstetrics and Gynaecology and Anaesthesia of a hospital in Moshi, Tanzania. KII and FGDs were held in a location convenient for the participants that would ensure privacy.

### Participants

All participants were healthcare providers and were chosen purposively, based on their involvement with maternity care and level of experience. Anaesthetic nurses and doctors working in the obstetric theatres were included to broaden the scope of the topic. FGDs were conducted with nurse-midwives to explore views of the same cadre and to enable triangulation of the data. Snowballing and opportunistic techniques were employed to identify healthcare providers and ensure participants were recruited sequentially until saturation was met.

### Topic guide

A topic guide was developed and piloted in the Kilimanjaro Christian Medical Centre, with five participants to refine and improve its quality. For example, the introduction was amended to ensure that the participants were aware that we sought to assess their general views and not their own personal experiences (if any) regarding the use of pain relief options (both pharmacological and non-pharmacological) during labour and after childbirth. The topic guide served as a flexible tool to facilitate the interviewer in obtaining the participants’ answers whilst ensuring that the interview remained on topic. The topic guide also acted as a cue to ask more probing questions to further understand participants’ knowledge and awareness. In addition to sociodemographic topics, the guide included four main subject areas: 1) knowledge and awareness of pain management options; 2) pain management practice in place; 3) perceptions and beliefs regarding different types of pain relief options (both pharmacological and non-pharmacological) and 4) recommendations to develop a comprehensive pain management service as part of better quality maternity care.

### Data collection

Prior to interview, all eligible participants were approached and given verbal and written information regarding the study including a brief overview of the research aims and interview questions. An interview appointment was then scheduled at a convenient time for the participant. All participants were interviewed in English, with the average interview lasting 30 min. All interviews were conducted face-to-face, recorded on a digital recording device and transcribed upon completion. Triangulation of results by method of data collection (KII and FGDs) and data sources (nurses-midwives, obstetric doctors, anaesthetic doctors and nurses) was used to increase validity [25]. All efforts were made to emphasise confidentiality to ensure the participants felt comfortable to provide honest answers. Interviewing participants with varied levels of experience from different departments in the hospital (antenatal clinic, labour ward, obstetric theatre, postnatal ward), provided a wide range of opinions and increased transferability.

### Analysis

Transcribed interviews were initially open coded and then reviewed by a second researcher for sense checking and to avoid bias. Codes were identified and grouped into categories by the first researcher and then reviewed by a second researcher, enabling the first abstraction of data [26]. Thematic framework analysis of the categories was then undertaken by the first researcher and independently by a second researcher. The separate results were then brought together and refined to agree on the key themes. This strengthened the results and helped to remove potential bias [27].

### Ethics

Full ethical approval was granted by the Liverpool School of Tropical Medicine, UK (LSTM14.025) and the Kilimanjaro Christian Medical College Research Ethics and Review Committee, in Moshi, Tanzania (N. 2047). Written informed consent was obtained from all participants of the study.

## Results

### Participants’ characteristics

Thirty-four healthcare providers participated, 24 in KII and 10 in two FGDs. Seventeen were doctors with different levels of experience (general junior doctor, specialist registrar, consultant) and 17 were midwives or nurse-midwives. Five healthcare providers were recruited from the Department of Anaesthesia and all worked in the obstetric theatres. Most participants were female (*n* = 24), aged between 26 and 35 years and had up to five years’ experience of providing maternity care.

### Emerging themes

The main emerging themes included: 1) enabling factors and opportunities; 2) barriers and challenges; and 3) context specific recommendations from healthcare providers on how to improve pain management.

### Enabling factors and opportunities

Factors facilitating the provision of pain relief for women in labour and after childbirth included: healthcare providers’ positive attitude to help women; awareness of both pharmacological and non-pharmacological various methods of pain relief; the common use of non-pharmacological pain relief methods during labour; and the routine use of opioids after Caesarean section.

#### Positive attitudes and awareness of pain relief

Most healthcare providers expressed positive attitudes regarding pain management during labour as part of routine maternity care (Table 1: Q.1, Q.2, Q.3). Many healthcare providers reported a good knowledge of non-pharmacological methods of pain relief including: comforting and counselling the women, provision of psychological support, back massage, breathing techniques, encouraging the presence of a companion in the early stages of labour and encouraging the mother to walk or have a bath (Table 1: Q.4, Q.5). Other methods such as transcutaneous electric nerve stimulation, yoga, acupuncture and water birth were mentioned (Table 1: Q.6, Q7). Most healthcare providers were also aware of both pharmacological options including oral medications (paracetamol, diclofenac and ibuprofen); opioids (codeine, tramadol, pethidine and morphine), nerve and pudendal block, and epidural and spinal anaesthesia. Only a few healthcare providers were aware of nitrous oxide inhalation as a pain relief option.

**Table 1:** Healthcare providers' quotes of enabling factors

THEME 1: Enabling factors for providing pain relief		
Sub-theme	Quote	
Positive attitudes	Q 1	“If there are pain relief drugs to give the mothers, let it come and be provided to the mothers so that they deliver peacefully. I could see that it’s better and good, and it will be helpful to the mother.” (Nurse-midwife, KII)
	Q 2	“I think [pain relief] should be part of management for those women who would wish to get that service.” (Specialist registrar in O&G, KII)
	Q 3	“I think it is good to provide [pain relief] because the labour pain is too much, and you go through the pain for hours and hours.” (Consultant Obstetrician and Gynaecologist, KII)
Knowledge of pain relief options	Q 4	“We are taught to allow the woman to walk; that could help to minimize the pain, also if there is a chance, the woman must be massaged on the back.” (Nurse-midwife, KII)
	Q 5	“You can do back rubbing, or […] ambulation, like sitting and walking or positioning; there is also partner involvement and […] bathing.” (Consultant Obstetrician and Gynaecologist, KII)
	Q 6	“I was reading in the internet that in some settings they have water birth delivery, that this is something to reduce pain.” (Midwife, KII)
	Q 7	“There is acupuncture, that can be used to relief the pain, but we don’t have anyone who is familiar with it.” (Midwife, FGD)
Current practice	Q 8	“To the mother in labour pain, I reassure her, I massage her […] I tell her to breath in and out to get relief and sometimes when she is tired, I encourage her; if she’s feeling like to bath, then I encourage her to bath.” (Nurse-midwife, KII)
	Q 9	“If she comes with a husband, or mother or mother-in-law, sometimes it is helpful, as you can call the relative […] to talk with the woman sometimes it helps to release the suffering.” (Specialist registrar in O&G, KII).
	Q 10	“Once the woman is contracting more and the cervix is not growing well with the contractions, we give buscopan or hyoscine, so at least the pain is a little bit reduced and the cervix is moving on.” (Midwife, KII)
	Q 11	“During labour, I think mostly we use buscopan and paracetamol.” (Specialist registrar in O&G, KII)
	Q 12	“For Caesarean sections, the protocol is pethidine for 24 h, thereafter we do give the paracetamol.” (Midwife, KII)
	Q 13	“When you have an episiotomy, it must be lignocaine, local analgesia and sutured.” (Consultant Obstetrician and Gynaecologist, KII)

#### Current use of pain relief

Most healthcare providers reported that they did routinely offer non-pharmacological options, such as counselling women about the nature and severity of labour pain and trying to provide psychological support and reassurance (Table 1: Q.8). Many healthcare providers commonly encouraged the support of a companion during the early stages of labour (Table 1: Q.9).

Many healthcare providers reported that they offered women paracetamol for pain; and buscopan and hyoscine were prescribed (erroneously) to distend the cervix (Table 1: Q.10, Q.11). Many healthcare providers reported that there was a protocol for management of pain after Caesarean section including pethidine for 24 h, followed by oral paracetamol and/or oral diclofenac according to the severity of pain (Table 1: Q.12). In cases of perineal or vaginal tears, or if an episiotomy was performed, some healthcare providers reported that an infiltration of lignocaine was recommended prior to suturing (Table 1: Q.13).

Within these themes, there was an underlying willingness of healthcare providers to provide better care, especially during labour, including to do more to alleviate pain and more frequently. However, there were barriers to the facilitation.

### Barriers and challenges

Barriers affecting the provision of pain relief options (both pharmacological and non-pharmacological) included: 1) health system barriers (lack of staff, equipment and protocols); 2) limited education and opportunity to practice pain relief methods (especially pharmacological); and 3) negative beliefs, fears and malpractice.

#### Health system barriers

Many healthcare providers highlighted the difficulty in providing ‘one-to-one’ individualised care for women because of the shortage of nurse-midwives compared to the high number of labouring women (Table 2: Q.14, Q.15). Although family members could stay with the woman in the early stages of labour in the antenatal ward, their presence was restricted in the labour room due to limited space and out of respect for the privacy of other women in labour room (Table 2: Q.16). Some healthcare providers were aware that labouring in water was a non-pharmacological option for pain relief but many explained that there were no facilities to implement this option in such settings.

**Table 2:** Healthcare provider quotes regarding barriers

THEME 2: Barrier to providing pain relief		
Sub-theme	Quote	
Shortage of staff	Q 14	“Let’s say we have four patients to monitor labour and all of them are in pain so you, you are the only one who is there in room 3 [pre-labour room], this is your location, you are the only healthcare provider, so how could you manage to help everyone; go and massage everyone who is in such pain, who is experiencing more pain?” (Midwife, KII)
	Q 15	“[…] I mean the shortage of nurses, or staff become a major challenge because you may find, maybe you’re allocated to a certain room, five or ten mothers are in labour, you are, you’re there by yourself …it becomes difficult.” (Nurse-midwife, FGD)
Privacy	Q 16	“We need to keep the privacy, and if you have a lot of relatives around and only a small curtain, there is no privacy to patients, no secret for them…we need more space.” (Nurse-midwife, KII)
Limited education and opportunity	Q 17	“I think maybe there a lack of trained personnel for [epidural], because it is not used here and no-one is experienced to teach us.” (Specialist registrar in Anaesthetics, KII)
	Q 18	“I think shortage of resources, especially we don’t have the catheters and monitors, you know for every patient you need a continuous tocographic machine for every patient, we don’t have these resources.” (Specialist registrar in O&G, KII)
	Q 19	“Teaching about pain management is not part of formal classes in medical school, because even when we learnt about labour, the slides on pain management was just one so, no, not much emphasis on pain management.” (Specialist registrar in O&G, KII)
	Q 20	“I’ve read about epidural, but I’ve no experience with epidural.” (Specialist registrar in O&G, KII)
	Q 21	“I know there are different methods of labour analgesia and epidural is one of them; but you can also give nitrous oxide but I’ve not much experience because I’ve just observed in some few centres abroad but I’ve not been trained on that.” (Consultant Obstetrician and Gynaecologist, KII)
Negative beliefs, fears, malpractices	Q 22	“There is a belief that this pain, we need to know how much pain this patient is experiencing at least at the beginning of the labour to be able to assess and evaluate the progress of labour.” (Junior doctor, KII)
	Q 23	“Once you give someone pethidine (she) may be dizzy, may feel like sleeping, so once someone is dizzy, and feel like sleeping all the time, how does she push?” (Midwife, KII)
	Q 24	“…the other thing is pain relief can cause harm to babies, they can sedate them, you’ll have an inactive baby, you can’t use it…” (Consultant Obstetrician and Gynaecologist, KII)
	Q 25	“…Whatever is available, like the opioids analgesics, they are not really recommended before a woman gives birth because that will also give respiratory depression to the babies, so before they deliver there is very little you can do…” (Specialist registrar in O&G, KII)
	Q 26	“They will go through labour and pain must be there so to deliver a baby, if there is no pain that means, there can’t be a baby without pain.” (Nurse midwife, KII)
	Q 27	“I’ve not practiced pain relief during labour because we assume that it should be there, and we take it as a normal, [but] of course it’s not normal but we take it as if every woman should experience this.” (Specialist registrar in O&G, KII)
	Q 28	“It happens sometimes the woman may get a tear; we normally give infiltration before starting repairing but some healthcare providers, they just stitch it without giving it, even if the mother is screaming, they just say “shut up her”, and just proceed, so it happens.” (Specialist registrar in Anaesthesia, KII)
	Q 29	“During episiotomy, sometimes they do not actually provide the lignocaine, local anaesthesia during the cutting but it is written in the book, it is written there.” (Nurse-midwife, FGD)
Limited availability of protocols	Q 30	“We don’t have a proper, pain management protocol for women who are delivering normally; we don’t give them analgesia.” (Specialist registrar in O&G, KII)
	Q 31	“For those of who had vaginal deliveries once they complain of severe pain we just give them diclofenac injection, maybe a start dose and observe; if the pain continues we give paracetamol.” (Junior doctor, KII)
	Q 32	“I have not seen a protocol anywhere, but we’ve just learned it from our senior that this is how we do things, this is how we manage this.” (Junior doctor, KII)

Many healthcare providers were aware of epidural medication but the limited number of trained anaesthetists and the lack of essential equipment (needles, catheters, drugs) meant this service was unavailable (Table 2: Q.17, Q.18). Most healthcare providers reported a lack of posters, leaflets and information materials regarding pain management options available for them or for the women.

#### Limited education and opportunity to practice pain relief methods

Various healthcare providers expressed a lack of specific education regarding different pain management (especially pharmacological options) during medical or midwifery undergraduate education (Table 2: Q.19). Healthcare providers expressed doubts regarding the efficacy of non-pharmacological methods.

With regards to pharmacological options, some healthcare providers explained that their experience was limited to the use of oral medication only; whereas training on nerve block, epidural and the use of nitrous oxide was not available (Table 2: Q.20, Q.21).

#### Negative beliefs, fears and malpractices

Some healthcare providers said they were concerned about missing important signs during labour if pharmacological pain relief was provided. The severity of pain during labour was considered an indicator of the progress of labour and, if removed, would hinder the correct evaluation of labour (Table 2: Q.22). Many healthcare providers explained that pharmacological pain relief interferes with the progression of labour and that all drugs (especially opioids) have detrimental side effects for the mother and/or the baby (Table 2: Q.23, Q.24, Q.25). In addition, almost half of all healthcare providers considered labour pain a ‘natural’ process, that does not require both pharmacological treatment or management (Table 2: Q.26). Moreover, most healthcare providers explained that it was the belief of the local community that labour pain must be present and that nothing can be done to relieve it (Table 2: Q.27).

Many healthcare providers mentioned the use of opioids was limited to women who had a Caesarean section. Some healthcare providers also reported that pharmacological pain relief was not part of the routine protocol for women who have had a vaginal delivery because it was assumed that these women did not have significant pain. Thus, oral paracetamol or diclofenac was given only if the woman requested it (Table 2: Q.30, Q.31).

Some healthcare providers reported witnessing colleagues suture perineal or vaginal tears without using lignocaine, despite its availability and the woman screaming in pain (Table 2: Q.28, Q.29). Misconception regarding the correct use of the options available (for example the use of buscopan to distend the cervix, fear of opioids, and limited use of non-pharmacological methods) was found to be a cross-cutting theme among the barriers.

### Recommendations on how to improve pain relief management

Many healthcare providers provided suggestions on how pain management (both pharmacological and non-pharmacological options) could be improved, by creating an enabling environment, providing antenatal education, and emphasising research and protocols. Most healthcare providers highlighted the need to expand the teaching of the correct use of pharmacological options including opioids and epidural analgesia as part of undergraduate and postgraduate education (Table 3: Q.33). Increasing the number of staff in the labour ward would allow the staff to improve non-pharmacological support for each woman and more nurses and clinicians should receive specialist training in anaesthesia (Table 3: Q.34, Q.35). Monthly staff educational meetings were suggested as opportunities to discuss new pharmacological methods and approaches, increasing awareness and discussion in the wider team of healthcare providers. Healthcare providers emphasised the need to develop context-specific pain management protocols (Table 3: Q.36). Some healthcare providers suggested further research to understand how best to provide pain relief care that meets a woman’s individual health needs (Table 3: Q.37). Some healthcare providers stressed the importance of educating women regarding the nature, progression and severity of labour pain during antenatal contacts (Table 3: Q.38).

**Table 3:** Healthcare provider quotes regarding solutions

THEME 3: Recommendations for providing pain relief		
Sub-theme	Quote	
Education for healthcare providers	Q 33	“I think we need to get education and to be educated on how and what specific pain relief should be given during labour pain, because sometimes you just start, you don’t know what to give. If we get that education I think it will be very helpful to us.” (Specialist registrar in Anaesthetics, KII)
	Q 34	“I suggest our health facility should liaise with the Government to promote more healthcare providers to go to anaesthetic school, because what we’re having here now it is a problem of shortage of this kind of profession.” (Nurse-midwife, FGD)
Increased staff numbers	Q 35	“We really need the number of staff to be the same as the number of women who are labouring.” (Specialist registrar in O&G, KII)
Cultural appropriate protocols	Q 36	“It’s better if everyone in the Department comes with something, then we discuss, we share, we know why we are doing this, in our setting, rather than copy from somewhere else.” (Specialist registrar in O&G, KII)
Research	Q 37	“I think we should do a study on women […] you can ask them what they really want during labour, if they really want analgesia or don’t. That will give us the way to set the service.” (Specialist registrar in Anaesthetics, KII)
Education for women	Q 38	“To improve our health education at the clinic, to tell the mothers, they should know that during labour they will feel pain; […] because having that in your mind you can tolerate the pain.” (Nurse, KII)

## Discussion

### Statement of principal findings

Most healthcare providers want to provide women with pain relief options (both pharmacological and non-pharmacological) but report feeling helpless in their attempts to support women due to a lack of staff and resources, limited education regarding the use of various methods of pain relief and the complex cultural context in which labour pain is considered. There are conflicting ideas between healthcare provider’s willingness to provide pain management during labour and the belief that this pain is natural and thus little can be done. Structural barriers limited implementation of pharmacological pain relief; whereas available options (opioids) were not routinely offered or used during labour due to fear of side effects for the mother and/or the newborn baby. Education (pre-service and in-service) would be helpful to develop healthcare providers’ confidence to offer more evidence-based pain relief options (both pharmacological and non-pharmacological) to all women during labour and after childbirth.

### Strengths of the study

To the best of our knowledge, this is the first study to use qualitative approaches to assess healthcare providers’ knowledge and attitudes regarding the provision of pain relief during labour and after childbirth in a low resource setting.

This study has highlighted key areas that need to be addressed to support the provision of routine pain relief options (both pharmacological and non-pharmacological) for women during labour and after childbirth. A range of healthcare providers who worked in different departments were interviewed resulting in a wide spectrum of responses. Interviews were also triangulated with information from FGDs improving the reliability of findings. All healthcare providers approached welcomed the discussion on routine pain relief during labour and after childbirth and were keen to contribute to ideas for solutions.

### Limitations of the study

This study population included mainly female healthcare providers who provide routine maternity care in a large teaching hospital in an urban setting and excluded cadres of healthcare providers who do not provide maternity care or work in a rural setting and may have alternative perspectives or different insights. Similarly, community-based healthcare providers may have different perceptions and experience. Their opinions would be important to ensure women have access to good quality of care including pain relief, in the community setting.

### How does this study relate to other literature?

In our study, healthcare providers routinely adopted non-pharmacological methods (breathing techniques, exercises, back massage, counselling and psychological support, companionship) as common pain management options; although most healthcare providers were doubtful regarding the efficacy of such methods. These approaches are used in many settings as a first line option, to improve the childbirth experience and increase women’s sense of control and participation in the decision-making process [4, 23, 28]. However, in our study a high workload and staff shortages (especially nurse-midwives) meant that it was not always possible to offer this support to all women at all time. In our study, simple oral medication (for example paracetamol) was the most common pharmacological pain relief option used during labour as a second line option, and opioids were reserved for women who have delivered by Caesarean section. However, in other settings, opioids are routinely offered to women in the early stages of labour [29–31]. Parenteral opioids are reported to be commonly prescribed by Indian obstetricians [10]. In Nigeria, two separate surveys confirmed the demand and acceptability of use of opioids for labouring women by various cadres of healthcare providers [32, 33] .

In our study, opioids were not offered to women during labour (due to concerns regarding the side effects and/or potential subsequent fetal compromise) and hyoscine and buscopan was given despite lack of evidence of benefit [34]. The reasons for this practice requires further study.

Some healthcare providers reported that they had witnessed colleagues suture perineal and vaginal tears without the use of local anaethetic, despite the availability of this pharmacological pain relief option. This practice is abuse against women, and goes against the principles of respecful maternity care, which requires healthcare providers to be gentle, respectful and establish effective communication with women to ensure they are informed regarding any interventions during labour and childbirth [14]. This sub-standard care or ‘malpractice’ must be addressed urgently [14]. Epidural analgesia during labour is widely available in well-resourced settings in high-income countries, but is often absent in the public sectors in low resource settings (as in our study) due to lack of an enabling environment. In India, the cost of the epidural service, the length of time required for monitoring and lack of staffing were the main barriers to the provision of epidural to women [35, 36]. In other studies, Indian obstetricians report a lack of education and training on epidural as a pharmacological pain management option in their training programmes [35, 36]. These challenges were similar to those reported in our study, where healthcare providers were not in a position to implement this service due to financial constraints [37] and a limited number of appropriately trained healthcare providers and/or anaesthetists (0.05 anaesthetists for every 100,000 people) [38].

Our study found that cultural beliefs have an influence on the attitudes of healthcare providers with many considering labour pains to be ‘natural’. In a similar study in Ethiopia, McCauley et al. [12] found that 24% of healthcare providers felt that pain should not be relieved and that labour pain was ‘natural’. In Bangladesh, 60% of healthcare providers believed strongly that ‘women should endure the natural pain of labour’ without pain relief being offered [39]. There is a need to better educate healthcare providers regarding the optimum use of available pharmacological options, including the physical and psychological consequences of protracted and unrelieved pain in women during labour and after childbirth [9, 14, 40]. We note that there are limited recommendations for the education, discussion, and provision for routine pain relief during labour and after childbirth in the recent updated WHO guidelines [41]. Amended antenatal care packages that include health education and health promotion regarding pharmacological and non-pharmacological pain relief options would be beneficial.

## Conclusion

Many healthcare providers in low resource settings do not routinely offer effective pharmacological pain relief during labour and after childbirth, despite some available resources. Most healthcare providers are open to helping women and improving quality of care during labour and after childbirth using an approach that respects a women’s culture wishes and beliefs. This study provides an understanding of the complexity of factors regarding the attitudes of healthcare providers to offer pain relief and provides recommendations to ensure pain management (both pharmacological and non-pharmacological) options are an integral part of maternity care. Women are increasingly accessing care during labour and there is a window of opportunity now to adapt and amend available maternity care packages to include comprehensive provision for pain relief as a component of quality of care.

## Abbreviations

FGD: Focus group discussion
KII: Key informant interviews
LSTM: Liverpool School of Tropical Medicine
O&G: Obstetrics and gynaecology
UK: United Kingdom
WHO: World Health Organization
